# Cognitive Impairment and Psychopathology Are Related to Plasma Oxidative Stress in Long Term Hospitalized Patients With Chronic Schizophrenia

**DOI:** 10.3389/fpsyt.2022.896694

**Published:** 2022-06-10

**Authors:** Man Yang, Jin Li, Haidong Yang, Linya Yan, Dongliang Liu, Lin Zhu, Xiaobin Zhang

**Affiliations:** ^1^Department of Psychiatry, The Fourth People's Hospital of Lianyungang, The Affiliated KangDa College of Nanjing Medical University, Lianyungang, China; ^2^Department of Psychiatry, Institute of Mental Health, Suzhou Psychiatric Hospital, The Affiliated Guangji Hospital of Soochow University, Suzhou, China; ^3^Department of Clinical Laboratory, The Fourth People's Hospital of Lianyungang, The Affiliated KangDa College of Nanjing Medical University, Lianyungang, China

**Keywords:** schizophrenia, oxidative stress, cognitive function, psychopathology, chronic

## Abstract

**Background:**

The present study aimed to examine whether plasma oxidative stress is associated with cognitive impairment in long term hospitalized patients with chronic schizophrenia.

**Method:**

Ninety-six chronic schizophrenia patients and 94 healthy unaffected subjects were enrolled. Plasma markers of oxidative stress, including malondialdehyde (MDA), manganese superoxide dismutase (MnSOD), catalase (CAT), and glutathione peroxidase (GSH-Px), were measured. Psychiatric symptoms and cognitive function were assessed with the Positive and Negative Syndrome Scale (PANSS) and the Repeatable Battery for the Assessment of Neuropsychological Status (RBANS), respectively.

**Results:**

Plasma MDA levels and MnSOD and GSH-Px activities were significantly lower in schizophrenia patients than in healthy controls (*P* < 0.001), while plasma CAT activity was higher than in healthy controls (*P* < 0.005). Cognitive scores on the RBANS and all of its five subscales (all *P* < 0.001) were significantly lower in schizophrenia patients than in healthy unaffected subjects. CAT and GSH-Px activities were positively correlated with the cognitive function scores corresponding to Visuospatial/Constructional abilities in the patient group (*r* = 0.298, 0.213, respectively, *P* < 0.05). Also, the multiple regression analysis revealed that CAT and GSH-Px activities were independent and separate contributors to the Visuospatial/Constructional index of the RBANS. Meanwhile, CAT activity was negatively correlated with general pathological symptoms (*r* = −0.307, Bonferroni corrected *P* = 0.008) and the total score of the PANSS domains (*r* = −0.299, Bonferroni corrected *P* = 0.012).

**Conclusion:**

Our results that the reduced of MDA level and the increased CAT activity in plasma in male patients with chronic schizophrenia suggest that redox imbalance may be associated with the pathophysiology of schizophrenia, and it can induce impaired cognition and psychiatric symptoms.

## Introduction

With an international lifetime prevalence of about 0.75%, schizophrenia (SZ) is a chronic and debilitating psychiatric disorder that is primarily characterized by the following three categories of symptoms: positive symptoms, negative symptoms, and more common cognitive deficits (1). Cognitive deficits, which are one of the core symptoms of SZ, include impairments in a range of domains such as attention, instantaneous or delayed memory, reasoning, executive function and processing speed, which contribute to poor treatment outcomes and disability in daily life, as well as reduced social functioning (2, 3). Indeed, a sizeable body of evidence has found that excessive reactive species and lowered antioxidant defenses may be implicated in SZ (4). However, the precise pathophysiological mechanisms underlying this distressing mental illness are not well understood, and further exploration is needed to improve long term clinical management (1).

Redox homeostasis and low or moderate concentrations of reactive oxygen species (ROS) play an important role in regulating normal physiological functions and maintaining various cellular signaling systems (5). Oxidative stress (OS) is a state of disequilibrium that occurs due to excessive production of ROS and an impaired antioxidant defense system (6, 7). Excessive ROS and impaired antioxidant defenses can induce damage to DNA and other biological components such as lipids, proteins, and enzymes, which can lead to cell dysfunction or even death in the brain, and furthermore contribute to the neuropathology of SZ (8). The end products of lipid peroxidation (LPO), especially malondialdehyde (MDA) assessment, is frequently chosen as indicators for oxidative stress in clinical studies (9). Meanwhile, antioxidant cellular defense is primarily enzymatic, involving superoxide dismutase (SOD), catalase (CAT) and glutathione peroxidase (GSH-Px), which are constitutively expressed in all tissues (10). Evidence of redox dysregulation has been reported in animal models of SZ. For example, Ozyurt et al. (11) found that oxidative stress levels increased in the prefrontal cortex in the MK-801-induced model. Furthermore, knockout mice with deficient GSH synthesis demonstrated parvalbumin inhibitory interneurons deficits in the anterior cingulate cortex (12).

Recently, a number of studies have shown that antioxidant enzyme levels are decreased, while LPO concentrations are increased in plasma, erythrocytes, cerebrospinal fluid, and different brain regions in first-episode, nonmedicated, medicated, and chronic SZ patients (13). Although these alterations may not constitute the basic cause of SZ, many studies have found that they may be one of the reasons for the declining course and poor prognosis observed in SZ. Therefore, OS parameters have been suggested as biomarkers of schizophrenia risk (14).

Whether the oxidative/anti-oxidative status relates to clinical status of SZ is of particular interest. Numerous studies have explored the relationship between ROS and the severity of different clinical dimensions in schizophrenia (3, 15), and found the complex changes of OS have been linked to cognitive impairment and psychiatric symptoms in both preclinical and clinical studies (16–18). Such as, serum SOD, GSH-Px were shown to correlate with overall severity in acute phase (19). And an animal study showed that antioxidant therapy significantly increased learning and memory by reducing antioxidant enzyme activities and in the prefrontal cortex and hippocampus in mice (20).

In view of the findings of the studies outlined above, several lines of evidence showed the disruption of the antioxidant defense system and increased oxidative injury in SZ patients (21). We hypothesized that an imbalance of oxidative stress in schizophrenic patients is related to the psychopathology in schizophrenia, including positive symptoms, negative symptoms and cognitive deficits. Therefore, our study will examine the relationship between plasma OS indicators and cognitive impairment and psychopathology in male schizophrenic patients with chronic long-term hospitalization of Chinese Han ancestry. This study aimed to: (1) probe whether altered ROS and antioxidant enzyme activities are observed in chronic medicated and long term hospitalized patients with SZ; and (2) examine the relationship between the markers of oxidative stress and cognitive function and psychiatric symptoms in SZ patients.

## Methods

### Subjects

Ninety-six inpatients (all male) with SZ were recruited from the regional psychiatric hospitals (i.e., Lianyungang Fourth People's Hospital, Ganyu District Rehabilitation Hospital, Donghai County Psychiatric Hospital, Guanyun County Psychiatric Hospital). All patients met the following inclusion criteria: (1) aged 20–60 years old, Han Chinese; (2) determined by the Structured Clinical Interview for the Diagnostic and Statistical Manual of Mental Disorders, 4th edition (DSM-IV), Patient Version (SCID-P); (3) at least 5 years of illness, and (4) had been treated with stable doses of neuroleptic drugs for at least 12 months. Antipsychotic medication, including both first- and second-generation antipsychotics, was converted into approximate daily dose milligram equivalents of chlorpromazine for each subject using standard guidelines (22). Exclusion criteria: Patients were comorbid neurological disorders such as mental retardation, dementia, epilepsy, degenerative disease, traumatic brain injury, and endocrine disorders such as thyroid dysfunction, diabetes mellitus, alcoholic, substance dependence.

Ninety-four age-, education-, and BMI-matched unaffected subjects (all male) were recruited from the local community in Lianyungang City. Unstructured interviews were carried out to assess current mental status and personal or family history of any mental disorder. None of the healthy subjects presented a personal or family history of psychiatric disorders. The complete medical history and the results of physical examination and laboratory tests were obtained from the patients and control subjects. Any subjects with major medical illnesses were excluded. None of the subjects met the criteria for drug or alcohol abuse/dependence. All subjects were of Han Chinese ethnicity, and recruited at the same period from the Lianyungang area. All subjects were explained by a psychiatrist fully with the research protocol and procedures and provided their written informed consent to participate in the study. The description of the study was tailored to maximize the understanding of the subject using language appropriate to the subject's level of comprehension, and emotional readiness. If the subject was willing to consent to participate in the study the researcher provided an in depth description to the subject and in certain instances, to their parents or guardians. The research protocol was approved by the Institutional Review Board of the Lianyungang Fourth People's Hospital.

### Plasma MDA Levels and Measurements of MnSOD, CAT, and GSH-Px Activity

Morning venous blood samples from both the SZ patients and healthy unaffected subjects were collected between 07.00 and 09.00 a.m. The collected anticoagulant blood samples were separated into plasma by centrifugation at 3,000 rpm for 15 min. All samples were stored at −80°C and refrigerated until further use. The activities of antioxidant enzymes (MnSOD, CAT, and GSH-Px) and levels of the oxidative stress product MDA were detected in accordance with a commercially available kit (A001-2, A007-1, A005, A003-1; Nanjing Jiancheng Bioengineering Institute, Nanjing, China) (6). The levels of GSH-Px were assessed using a colorimetric method, MDA analysis used a thiobarbituric acid method, MnSOD involved hydroxylamine method and CAT was determined using a visible light method according to the manufacturer's instructions.

MnSOD and CAT activities were expressed as a unit per milliliter plasma (U/ml), the plasma MDA level was expressed as MDA milli-mole per liter plasma (mmol/L), and the activity of GSH-Px was expressed as nanomole per milliliter plasma (nmol/ml).

### Cognitive Tests

In this study, the cognitive ability of SZ patients and healthy controls was measured with the Repeatable Battery for the Assessment of Neuropsychological Status (RBANS) which was previously translated to produce the RBANS Chinese version (23). RBANS has shown good reliability and validity in patients with psychosis and takes approximately 30 min to complete, which means that this instrument is practical and feasible to use in practice.

The cognitive domains included five age-adjusted index scores (i.e., the Immediate Memory Index, Visuospatial/Constructional Index, Language Index, Attention Index, and Delayed Memory Index) and a total score. The original scores of the neuropsychological variables were converted to T-scores using the available criteria outlined in the corresponding manuals. A higher RBANS score indicates better cognitive functioning.

### Clinical Measures

The psychopathology of the patients was assessed with the Positive and Negative Syndrome Scale (PANSS) (24) on the day of blood drawing, and conducted independently by two psychiatrists, who had simultaneously attended a training session in the use of the scale before the study began. After training, to ensure the consistency and reliability of the ratings produced during the study, we conducted two interviews with the PANSS scale for one patients by two psychiatrists separately. And a correlation coefficient >0.8 was maintained for inter-rater reliability on the PANSS total score at repeated assessments. In this study, the original PANSS consisted of five factors (25), namely, positive factor (composed of P1, P3, P5, G9), negative factor (composed of N1, N2, N3, N4, N6, G7), disorganization factor (composed of P2, N5, G11), excitement factor (composed of P4, P7, G8, G14), and depressive factor (composed of G2, G3, G6) (15).

### Statistical Analysis

First, we examined the normality of the data using Q–Q plots, the Kolmogorov–Smirnov test, and the Shapiro–Wilk test. Demographic and clinical variables of the SZ patients and healthy controls were compared with t-tests for normally distributed continuous variables and chi-squared tests for categorical variables. Mann–Whitney *U*-tests were then performed to evaluate non-normally distributed variables. We compared RBANS scores between SZ patients and controls using the analysis of covariance. The effect of age, education, BMI, and smoking status was tested by adding these variables to the analysis model as covariates. The relationships between the variables were assessed with Pearson's or Spearman's product moment correlation coefficients. Bonferroni corrections were applied to each test to adjust for multiple testing. For example, the Bonferroni corrected *P-*value for each RBANS score is the original *P-*value ×6. Moreover, multivariate regression analyses were performed to assess possible associations between OS factors (MDA, MnSOD, CAT, and GSH-PX) and RBANS, while adjusting for various potentially confounding variables including age, education, and smoking status in both the patient and control groups, as well as clinical variables in the patient group, such as the PANSS total and index scores, age of onset, course of disease, and antipsychotic treatment. The Statistical Product and Service Solutions (SPSS) version 22.0 (IBM, USA) was used for all statistical analysis. Data are presented as mean ± standard deviation (SD). All *P*-values are two-tailed and the significance level was set at 0.05.

## Results

### Demographic Data

Clinical and demographic characteristics for the SZ patients and healthy unaffected subjects are presented in Table 1. Patients and control groups showed no differences in age, education, smoking status, and body mass index (BMI; all *P* > 0.05). When the relationship of all of the parameters was then examined separately in the patient and control groups, age, education, BMI and smoking status were not associated with the ROS index MDA level or with the antioxidant stress index for MnSOD, CAT, and GSH-Px activities (all *P* > 0.05). Moreover, in the patient group, age of onset of psychosis, duration of illness, and dose of antipsychotic drugs were not correlated with the MDA level or with MnSOD, CAT and GSH-Px activities (all *P* > 0.05).

**Table 1:** Demographic and clinical characteristics of schizophrenia patients and healthy controls (Mean ± SD).

	**Patients**	**Healthy controls**	**χ^2^ or**	***P*** **-Value**
	**(*n* = 96)**	**(*n* = 94)**	***t*** **or *z***	
Age (years)	40.4 ± 9.6	40.6 ± 9.5	-0.18	0.855^a^
Education (years)	9.3 ± 3.2	9.6 ± 3.2	-0.34	0.734^c^
BMI	24.4 ± 3.8	25.3 ± 2.9	-1.83	0.069^a^
Smoker/nonsmoker	49/47	41/53	1.05	0.305^b^
Age of onset (years)	26.7 ± 8.8			
Duration of illness (months)	160.4 ± 93.6			
Daily AP dose (mg/day) (CPZ equivalent)	688.7 ± 313.2			
PANSS total score	58.4 ± 14.2			
P subscore	10.9 ± 4.2			
N subscore	18.2 ± 7.0			
G subscore	29.2 ± 6.1			

*BMI, body mass index; AP, antipsychotic; CPZ, chlorpromazine; PANSS, Positive and Negative Symptom Scale; P, PANSS positive symptom subscale; N, PANSS negative symptom subscale; G, PANSS general psychopathology subscale*.

a *Means Independent samples t-test*.

b *Means χ^2^ test*.

c *Means Mann–Whitney U-test*.

### Plasma Oxidative Stress Parameters

Table 2 summarizes oxidative stress levels in blood samples from the SZ patients and healthy controls. Chronic patients had significantly lower levels of MDA, MnSOD activity, and GSH-Px activity compared with healthy unaffected subjects, but higher CAT activity. This difference remained significant after covarying for age, education, BMI, and smoking status.

**Table 2:** Oxidative stress levels of schizophrenia patients and healthy controls (Mean ± SD).

	**Patients**	**Healthy controls**	**χ^2^ or**	***P*** **-Value**
	**(*n* = 96)**	**(*n* = 94)**	***t*** **r *z***	
MDA (mmol/L)	6.2 ± 3.1	11.3 ± 2.8	−9.14	^**^0.000^b^, ^c^
MnSOD (U/ml)	4.2 ± 2.0	8.6 ± 2.8	−9.71	^**^0.000^b^, ^c^
CAT (mmol/L)	3.2 ± 0.6	2.8 ± 0.8	−2.38	^*^0.017^b^, ^c^
GSH-PX (nmol/ml)	67.6 ± 13.4	84.4 ± 19.8	−6.831	^**^0.000^a^, ^c^

a *Means Independent samples t-test*.

b *Means Mann–Whitney U-test*.

c *Means after controlling for age, education, BMI, smoking status, the difference in MDA, MnSOD, GSH-Px still remained significant*.

* *P < 0.05;*

** *P < 0.01*.

#### Cognitive Performance in SZ and Healthy Controls

The mean and standard deviation of the RBANS total and index scores of 96 patients and 94 normal unaffected subjects are shown in Table 3. The patients performed worse in total score and all subscales (all *P* < 0.001 Bonferroni correction, *P* < 0.001). After controlling for age, education, BMI, and smoking status, the differences remained significant for the RBANS total score and subscales (all *P* < 0.001).

**Table 3:** Total and index scores on the RBANS in schizophrenia vs. controls.

**Index**	**Schizophrenia**	**Controls**	**Adjusted**	**Adjusted**
	**(*n* = 96)**	**(*n* = 94)**	***F*** **^a^**	***P-*** **value**
Immediate memory	50.0 ± 16.4	83.8 ± 16.9	187.14^a^	^*^ <0.001
Attention	81.3 ± 14.3	108.1 ± 14.3	174.60^a^	^*^ <0.001
Language	72.2 ± 13.4	97.6 ± 11.1	203.37^a^	^*^ <0.001
Visuospatial/constructional	68.7 ± 15.2	89.9 ± 14.2	95.83^a^	^*^ <0.001
Delayed memory	53.0 ± 15.8	85.5 ± 16.9	178.16^a^	^*^ <0.001
Total	57.8 ± 11.0	88.8 ± 12.6	343.85^a^	^*^ <0.001

* *P < 0.01*.

a *Means that F value was controlled for age, smoking, BMI, and education*.

### Association Between OS Markers and Psychotic Symptoms

Subsequently, tests were carried out to assess the correlation between the clinical variables and oxidative stress levels in SZ patients. No significant correlation was found between OS indexes and the PANSS total or its subscale scores (all *P* > 0.05), except in the case of CAT activity. We observed a negative association between CAT activity and the PANSS total score (*r* = −0.299, Bonferroni corrected *P* = 0.018; Table 4).

**Table 4:** Relationships between oxidative stress markers and psychotic symptom in patients^a^.

		**MDA**	**MnSOD**	**CAT**	**GSH-PX**
		**(mmol/L)**	**(U/ml)**	**(mmol/L)**	**(nmol/ml)**
Positive factor	*r*	−0.159	0.032	−0.114	0.005
	*P*	0.123	0.756	0.268	0.961
	*P* ^b^	0.728	4.536	1.608	5.766
Negative factor	*r*	−0.106	−0.118	−0.135	−0.054
	*P*	0.305	0.154	0.191	0.600
	*P* ^b^	1.83	0.924	1.146	3.600
Cognitive factor	*r*	−0.128	−0.192	−0.138	−0.002
	*P*	0.214	0.061	0.179	0.981
	*P* ^b^	1.284	0.366	1.074	5.886
Excited factor	*r*	−0.113	−0.034	−0.223	−0.129
	*P*	0.271	0.741	0.029	0.210
	*P* ^b^	1.626	4.446	0.174	1.260
Depression factor	*r*	−0.064	0.127	−0.111	−0.085
	*P*	0.538	0.217	0.282	0.412
	*P* ^b^	3.228	1.302	1.692	2.472
PANSS total score	*r*	−0.212	−0.060	−0.299	−0.014
	*P*	0.038	0.560	0.003	0.891
	*P* ^b^	0.228	3.360	0.018^*^	5.346

a *Means Spearman product moment*.

b *Means Bonferroni correction was applied in the associations between biomarkers and psychotics symptoms*.

* *P < 0.05*.

**Figure 1:**
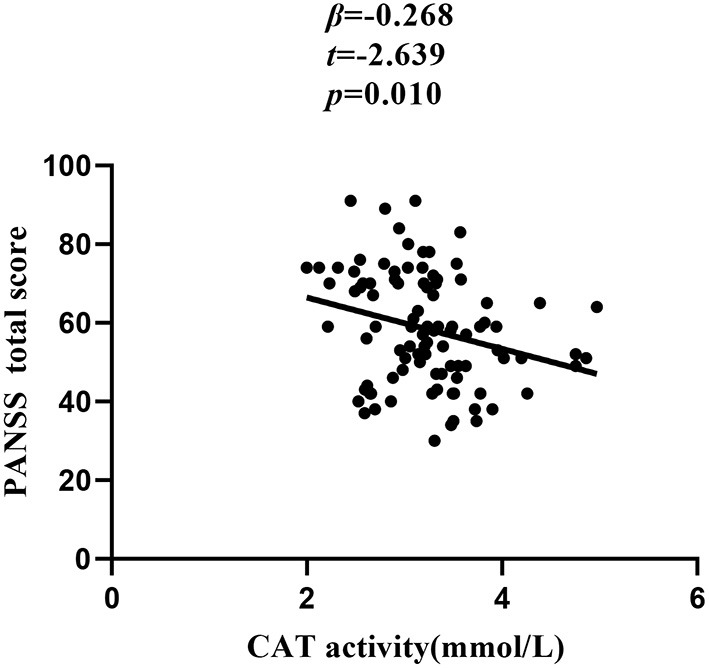
Correlation between CAT activity and PANSS total score in chronic schizophrenia patients.

Further stepwise multiple regression analysis with age, education, smoking, BMI, age of onset, dose of antipsychotic treatment, and duration of illness and oxidative stress marker as covariates identified CAT (β = −0.268, *t* = −2.639, *P* = 0.010) as influencing factor for the PANSS total score (Figure 1).

### Association Between OS Markers and Cognitive Performance

**Figure 2:**
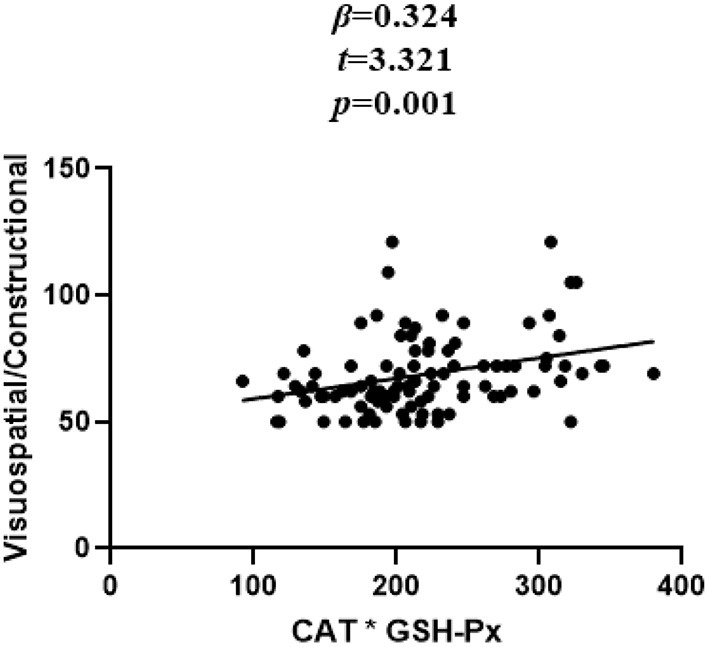
Correlation between plasma CAT^*^GSH-Px and Visuospatial/Constructional score in chronic schizophrenia patients.

**Figure 3:**
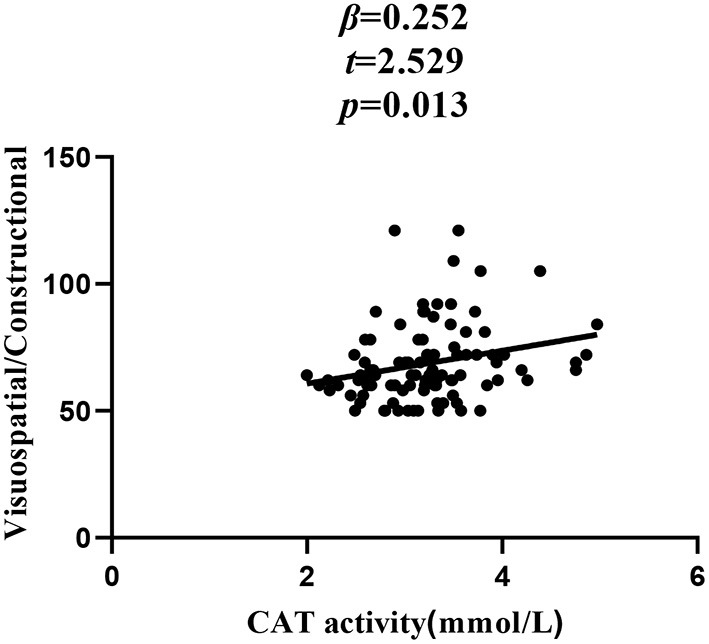
Correlation between plasma CAT activity and Visuospatial/Constructional score in chronic schizophrenia patients.

**Figure 4:**
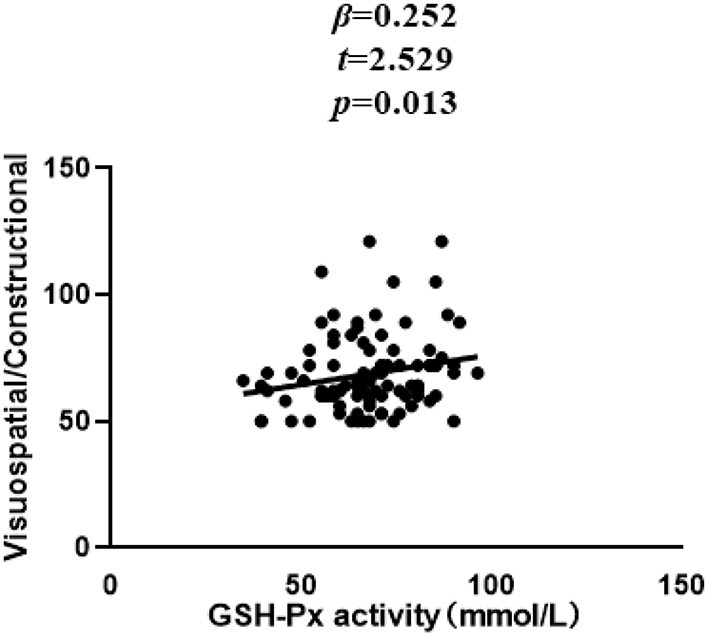
Correlation between plasma GSH-Px activity and Visuospatial/Constructional score in chronic schizophrenia patients.

In patients with SZ, a positive correlation was found between the Visuospatial/Constructional RBANS index and CAT (*r* = 0.298, *P* = 0.003) and GSH-Px (*r* = 0.213, *P* = 0.037) activities. However, after Bonferroni correction, the latter correlation remained significant only in the case of CAT activity (*r* = 0.298, Bonferroni corrected *P* = 0.018). For healthy unaffected subjects, no association was found between the OS markers and any cognitive index or total score of the RBANS (all *P* > 0.05). Further, stepwise multiple regression analysis that CAT ^*^ GSH-Px (β = 0.324, *t* = 3.321, *P* = 0.001; Figure 2), CAT (β = 0.252, *t* = 2.529, *P* = 0.013; Figure 3) and GSH-Px (β = 0.211, t= 2.088, *P* = 0.039; Figure 4) were independent contributors to the Visuospatial/Constructional Index of RBANS, after controlling for age, education, smoking, BMI, age of onset, dose of antipsychotic treatment, and duration of illness. But there was no significant association between OS marker (except CAT and GSH-Px) and RBANS total score or index scores in patients (all *P* > 0.05).

## Discussion

Overall, the main findings of the present study on long term medicated SZ inpatients are as follows: (1) compared with healthy unaffected subjects, significantly lower plasma MDA levels were found in SZ patients who were administered psychotropic agents and, with the exception of plasma CAT activity, MnSOD and GSH-Px antioxidant enzyme activities were decreased in chronic SZ patients; (2) plasma CAT activities were negatively correlated with clinical symptoms; (3) all of the RBANS test scores were significantly lower in long term hospitalized patients with chronic SZ than in healthy unaffected subjects, and (4) in patients with chronic SZ, plasma GSH-Px and CAT plasma activity concentrations were positively and independently correlated with deficits in Visuospatial/Constructional abilities after controlling for covariates (i.e., age, education, age of onset, duration of psychosis, PANSS total score, and PANSS subscale scores).

We found significantly lower lipid peroxidation product plasma MDA levels in the patient group, which is contrary to several studies which described elevated MDA levels in chronic medicated SZ patients compared with healthy unaffected subjects (21). To the best of our knowledge, this study is the first to report lower plasma MDA levels in chronic SZ patients who were long term medicated. However, decreased plasma thiobarbituric acid-reactive substance (TBARS) levels (26) and plasma lipid peroxidation (LPO) levels (18) with antipsychotic treatment have been reported. One possible explanation for these differences may be variations in medication use, whereby treatment with atypical antipsychotics was associated with a reduction of oxidative stress, which causes damage to all biomolecule types, including lipids (27). In our study of OS biomarkers, the results revealed that, in contrast to reduced plasma MnSOD and GSH-Px activities, the activity of the important antioxidative enzyme CAT was elevated in the stable SZ cohort compared with normal ranges, after controlling for covariates. Few previous studies involving chronic and stable SZ patients have reported elevated plasma CAT (18) and little research evidence has detected lower plasma CAT levels (28). Nevertheless, our findings are consistent with a meta-analysis which reported that, unlike GSH-Px, CAT seemed to be a state-related marker, as activities were significantly lower in first-episode psychosis, while they were increased in stable patients with antipsychotic treatment, and subsequently decreased in chronic patients (29). In chronic patients after an acute episode and long term treatment, it is likely that increased CAT activity within the antioxidant defense system will neutralize free radicals, preventing potential damage from maintained oxidative stress. In this sense, the finding of decreased plasma MDA levels in our sample might be the result of this efficient response.

In addition, we found that plasma CAT activity was negatively associated with the PANSS total score, which is in agreement with a case-control study that observed a negative correlation between plasma CAT activity and the general pathology scores and total scores, as assessed by the PANSS (7). However, we did not replicate previous findings of a positive correlation between MnSOD (26) and GSH-Px (30) and severity of psychopathology symptoms. Therefore, the association between the altered oxidative stress indexes and clinical symptoms provides further support for the view that oxidative stress constitutes a risk factor for the severity of clinical symptoms in SZ (31). Comparable to most other studies, we found reduced plasma MnSOD and GSH-Px activities in chronic SZ patients (21, 32). In contrast, other investigators found elevated levels of plasma GSH-Px and SOD in chronic medicated patients with SZ (10), and some studies failed to find any difference in serum GSH-Px activity (9) between patients and controls. In the current study, lower plasma MnSOD and GSH-Px activities in the SZ patients appear to indicate that impaired oxidative defenses are implicated in this disorder (21). However, several factors may have contributed to these discrepancies in research findings, such as duration of illness, clinical status of the patients, type of antipsychotic drugs taken, dosage and length of administration, or biological and ethnic heterogeneity, lifestyle and dietary patterns (3). Typical antipsychotics could decrease oxidative stress and alter antioxidant enzyme levels in animal studies and in schizophrenic patients (33, 34), and the mechanisms of these may be up-regulating SOD gene expression and increasing 5-hydroxyindoleacetic acid (5-HIAA), which is an excellent scavenger of hydroxyl and superoxide radicals (34). Despite heterogeneity among these clinical studies in SZ, the observed abnormalities in antioxidant enzyme activities indicated a weakness in the efficiency of antioxidant defenses, leading to oxidative toxicity. Consequently, in chronic SZ patients, we observed disrupted redox reactions. A major hypothesis suggest that unbalanced oxidative stress state can result in OS-induced neurotoxicity or neurodegeneration in the central nervous system in SZ and these changes might lead to some of the structural and functional alterations in the brain (35). Overall, it appears that oxidative stress may have a close reciprocal relationship in the development of SZ, though the mechanisms are not fully understood and clearly warrants further investigation.

Cognitive deficits are a strong predictor of poor life outcomes in SZ patients (36). The chronic long term hospitalized SZ patients who participated in this study had significantly lower cognitive performance than healthy unaffected subjects on all of the five RBANS subscales (37). These deficits are consistent with the majority of studies that assessed cognitive performance in SZ patients, suggesting that cognitive deficits are a core feature of schizophrenia (15, 38). However, despite extensive research, the etiology of the cognitive deficits observed in remains very poorly understood (39).

We found a positive correlation between plasma GSH-Px and CAT activities and the Visuospatial/Constructional Index of cognitive function. However, previous studies, such as Zhang et al. (3), produced opposing findings, and proposed that the MnSOD antioxidant index was decreased, but it seems constituted a risk factor for impaired cognitive function in SZ, particularly in respect to attention and total cognitive performance. Moreover, Wei et al. (40) found that serum CAT activity was negatively associated with the Wisconsin Card Sorting Test (WCST) error scores. However, several animal studies also support the hypothesis that antioxidant supplementation can improve cognitive functioning (41, 42), which furthermore highlights oxidative stress as a risk factor for cognitive impairment in SZ. In this study, the regression analysis revealed an additional interesting finding: after adjusting for conventional risk factors, the interactions of plasma GSH-Px activity and CAT activity were linked to the Visual-Spatial attention aspect of cognitive function, which suggests that the imbalance in antioxidant defenses may critically contribute to cognitive impairment in SZ. It has been reported that neurotoxicants can lead to an imbalance between pro-oxidant elements (ROS) and antioxidants (reducing elements) by modulating the *N*-methyl-d-aspartic acid (NMDA) receptor in the hippocampus in the brain (43). The imbalance between defensive elements and ROS causes neuronal cell death in the synaptic regions of the hippocampus, and in this process, ROS dominates antioxidant factors such as GSH-Px, glutamine synthetase (GS), GSH, CAT, SOD, and brain-derived neurotrophic factor (BDNF), which ultimately leads to cognitive dysfunction (44). Dean reported that glutathione deficiency induces oxidative damage to short term spatial memory in rats and mice in the Y-maze test (45). Meanwhile, it was demonstrated that CAT and GSH-Px expression in the brain tissue were altered endogenously in the rat hippocampus affected by nutritional vitamin A deficiency, which can impair the temporal orchestration of hippocampal daily cognitive performance (16). Finally, in terms of cognitive functioning, as oxidative stress biomarkers have been found to be related to the cognitive performance of healthy elderly subjects and those with Alzheimer's disease (46), we can conclude that there appears to be a relationship between oxidative stress parameters and SZ in this study. While the mechanisms underlying the association between redox imbalance and impaired cognition remain unknown, the role of oxidative stress in both the pathogenesis of SZ and cognitive dysfunction may indicate one of the intricate links.

It should be noted that this study had several limitations. First, the relatively small sample size might not be completely representative of all SZ patients. Second, this pilot study detected oxidative stress in SZ using representative markers for the antioxidant defense system, such as enzymatic (MnSOD, CAT, GSH-Px) and lipid peroxidation products (MDA). For a more in-depth understanding of oxidative stress in relation to SZ and related cognitive impairment, future studies could examine a larger cohort to systemically profile all of the primary antioxidant enzymes that act in concert, including CuZnSOD, T-NOS, POD, and T-AOC, as well as antioxidant non-enzymatic molecules and other ROS, such as superoxide, nitric oxide, hydrogen peroxide, and free radicals. Third, it is still uncertain whether the OS parameters in plasma may reflect similar changes in the central nervous system. Fourth, the patient group differed from the control group in terms of their illness and the administered drug treatment. It is difficult to definitively determine whether the illness or the drugs produced the differences in the antioxidants and MDA levels investigated in the present study, particularly given that the patients were chronically ill and medicated. Moreover, other factors, such as exercise and diet/nutrition, are known to affect oxidative markers and these were not controlled for in the present study, which could have influenced the observed between-group differences. Unfortunately, we did not collect dietary, lifestyle, or exercise data. It is a fact that comorbidity and substance abuse are very prevalent in SZ. Therefore, we should enroll these subgroup patients in our future research. Given the fact that gender differences exist in cognitive domains and oxidative stress parameters in both schizophrenia patients and controls (47–49), we included male subjects in this study only. Despite these limitations, the current study has some noteworthy strengths. The sample included an age- and education-matched control group, and a large number of confounding factors were considered in the multiple regression analyses.

## Conclusions

In conclusion, our preliminary study demonstrated redox reaction imbalance in chronic medicated SZ patients, compared with healthy matched controls. In addition, cognitive deficits, especially impaired visual-spatial abilities, may be associated with abnormal CAT and GSH-Px activities. Taken together, we speculate that OS may modulate specific aspects of cognitive function that are relevant to chronic medicated patients with SZ. The mechanisms underlying the relationship between OS and SZ warrant further exploration. Although we are still far from determining valid and specific biomarkers of this heterogeneous illness, a biological approach to this type of research continues to lead us toward promising new horizons in the field of diagnostic, prognostic, and therapeutic methods of clinical practice.

## Data Availability Statement

The original contributions presented in the study are included in the article/supplementary files, further inquiries can be directed to the corresponding author.

## Ethics Statement

The research protocol was approved by the Institutional Review Board of the Lianyungang Fourth People's Hospital. The patients/participants provided their written informed consent to participate in this study.

## Author Contributions

XZ, MY, and JL were responsible for study design, statistical analysis, and writing of the manuscript. LZ was responsible for laboratorial analysis. HY, LY, and DL were responsible for recruiting the patients, performing the clinical rating, and collecting the samples. All authors have contributed to and have approved the final manuscript.

## Funding

This study was supported by grants from Suzhou Clinical Key Disciplines for Geriatric Psychiatry (SZXK202116), Key Diagnosis and Treatment Program of Suzhou (LCZX201919), the Jiangsu Province Social Development Project (BE2020764), Suzhou Clinical Medical Center for mood disorders (No. Szlcyxzx202109) and General program of Lianyungang Health Committee (No. 202130). The finding sources of this study had no role in study design, data collection and analysis, decision to publish, or preparation of the article.

## Conflict of Interest

The authors declare that the research was conducted in the absence of any commercial or financial relationships that could be construed as a potential conflict of interest.

## Publisher's Note

All claims expressed in this article are solely those of the authors and do not necessarily represent those of their affiliated organizations, or those of the publisher, the editors and the reviewers. Any product that may be evaluated in this article, or claim that may be made by its manufacturer, is not guaranteed or endorsed by the publisher.
